# Contacting of authors by systematic reviewers: protocol for a cross-sectional study and a survey

**DOI:** 10.1186/s13643-017-0643-z

**Published:** 2017-12-08

**Authors:** Reint Meursinge Reynders, Luisa Ladu, Nicola Di Girolamo

**Affiliations:** 1 Department of Oral and Maxillofacial Surgery, Academic Medical Center, University of Amsterdam, Meibergdreef 9, 1105 AZ Amsterdam, The Netherlands; 2 Private practice of orthodontics, Via Matteo Bandello 15, 20123 Milan, Italy; 3 EBMVet, Via Sigismondo Trecchi 20, 26100 Cremona, CR Italy

**Keywords:** Systematic review, Meta-analysis, Methodology, Author contact, Replying author, Risk of bias, Missing data, Reporting, Cochrane Collaboration, Email address, Contact data

## Abstract

**Background:**

Synthesizing outcomes of underreported primary studies can pose a serious threat to the validity of outcomes and conclusions of systematic reviews. To address this problem, the Cochrane Collaboration recommends reviewers to contact authors of eligible primary studies to obtain additional information on poorly reported items. In this protocol, we present a cross-sectional study and a survey to assess (1) how reviewers of new Cochrane intervention reviews report on procedures and outcomes of contacting of authors of primary studies to obtain additional data, (2) how authors reply, and (3) the consequences of these additional data on the outcomes and quality scores in the review. All research questions and methods were pilot tested on 2 months of Cochrane reviews and were subsequently fine-tuned.

**Methods for the cross-sectional study:**

Eligibility criteria are (1) all new (not-updates) Cochrane intervention reviews published in 2016, (2) reviews that included one or more primary studies, and (3) eligible interventions refer to contacting of authors of the eligible primary studies included in the review to obtain additional research data (e.g., information on unreported or missing data, individual patient data, research methods, and bias issues). Searching for eligible reviews and data extraction will be conducted by two authors independently. The cross-sectional study will primarily focus on how contacting of authors is conducted and reported, how contacted authors reply, and how reviewers report on obtained additional data and their consequences for the review.

**Methods for the survey:**

The same eligible reviews for the cross-sectional study will also be eligible for the survey. Surveys will be sent to the contact addresses of these reviews according to a pre-defined protocol. We will use Google Forms as our survey platform. Surveyees are asked to answer eight questions. The survey will primarily focus on the consequences of contacting authors of eligible primary studies for the risk of bias and Grading of Recommendations, Assessment, Development and Evaluation scores and the primary and secondary outcomes of the review.

**Discussion:**

The findings of this study could help improve methods of contacting authors and reporting of these procedures and their outcomes. Patients, clinicians, researchers, guideline developers, research sponsors, and the general public will all be beneficiaries.

**Electronic supplementary material:**

The online version of this article (10.1186/s13643-017-0643-z) contains supplementary material, which is available to authorized users.

## Background

Reporting in research studies is often suboptimal [1–3]. Synthesizing the outcomes of poorly reported studies can pose a serious threat to the validity of systematic reviews and healthcare guidelines [4–7]. The Cochrane Collaboration therefore recommends reviewers to contact authors of eligible studies included in the review and to request additional information on poorly reported items in these studies [8, 9]. In new Cochrane intervention reviews, we will quantify issues such as (1) how reviewers report on procedures and outcomes of contacting authors to obtain additional information, (2) how contacted authors reply, and (3) the consequences of the additional obtained data for the risk of bias and Grading of Recommendations, Assessment, Development and Evaluation (GRADE) scores [10] and for the primary and secondary outcomes of the review.

Poor reporting does not permit an adequate assessment of how a study was conducted and whether systematic error could have influenced outcomes. Various research papers have assessed these issues and have confirmed that reporting of trial findings is often incomplete and biased [1–3]. Adverse events are also often underreported and can lead to misconceptions on the safety of interventions [11–13]. Addressing incomplete reporting of primary studies is particularly important for systematic reviews, because reporting issues can influence the conclusions of such studies [4–7] and could raise serious doubts whether the outcomes of a systematic review can be trusted. This is a problem for a variety of stakeholders including patients, clinicians, researchers, health care policymakers, insurance companies, pertinent healthcare industries, research sponsors, or others.

To systematically tackle inadequate reporting, the Enhancing the Quality and Transparency Of health Research (EQUATOR) Network seeks to improve reporting by promoting the wider use of key reporting standards for a variety of study designs [14]. Although these guidelines are endorsed by many journals, improvements of reporting in research articles are still small or insufficient [15, 16]. Contacting the original authors of a research study by systematic reviewers to obtain additional research data is another strategy for dealing with incomplete reporting [8, 9, 17]. Such methods have provided important research data that have permitted systematic reviewers to (1) assess the eligibility of studies [18, 19], (2) assess conflicts of interest [20], (3) obtain research protocols [21], (4) assess the study design [19], (5) obtain information on groups of participants [22], (6) obtain outcomes [19], (7) calculate missing statistics [18, 23, 24], (8) assess sample sizes [25], (9) assess information on blinding of participants and personnel [26], (10) fine-tune risk of bias assessments [23, 24], and (11) assess the risk of selective outcome reporting [21]. Contacting of study authors is defined as “Highly desirable” according to the Methodological standards for the conduct of new Cochrane Intervention Reviews (MECIR) [9]. To scrutinize this issue, we will ask a series of questions on contacting of authors in these “MECIR guided” systematic reviews such as (1) how the reviewers report on contacting of authors of eligible studies, (2) the prevalence of reviews that contacted studies, (3) the validity of author contact data, (4) how contacted authors reply, (5) how reviewers report on the obtained additional data, and (6) what the consequences of these additional data are for the risk of bias scores of the eligible primary studies and for the GRADE [10] scores and the primary and secondary outcomes of the review. We will also quantify the prevalence of “Unclear” risk of bias scores in the eligible studies in these new Cochrane intervention reviews, because this statistic could be an indicator of the need to contact authors of primary research studies to obtain additional information.

To assess what has been published on these issues, we conducted scoping searches in the following electronic databases: Pubmed (MEDLINE), EMBASE (Ovid), and Cochrane Central Register of Controlled Trials (CENTRAL) [27]. The “Related Articles” feature in PubMed was also used to find additional studies. These procedures identified various publications on contacting of authors, but little has been published on our research questions [17, 28–34]. For example, (1) Two papers [17, 28] discussed the importance of contacting authors, (2) Schroll et al. [29], Selph et al. [30], and Van Driel et al. [31] asked different research questions on contacting authors, (3) Wolfe et al. [32] did not ask most of our questions, focused exclusively on missing drug trial data, and used different methodology, (4) Young et al. [33] assessed the effects of different methods to obtain additional data, but did not ask our research questions (3), and (5) Mullan et al. [34] showed that procedures and outcomes of contacting authors in top medical journals and Cochrane reviews were suboptimal but this study included only a small number of Cochrane reviews (*n* = 54), assessed data of more than 10 years ago, and did not cover most of our research questions. We also conducted a cross-sectional and a survey pilot study in 2 months of Cochrane intervention reviews (July and August 2015), which (1) confirmed the importance of addressing our questions, (2) showed the need to use both a cross-sectional study and a survey to adequately address these questions, and (3) identified at least one “Unclear” risk of bias domain in most of the eligible studies of these reviews. The published evidence on the importance of obtaining additional research information from original investigators, the knowledge gaps in the literature on our research questions, and the outcomes of our pilot tests all demonstrated the need for undertaking this study.

## Objectives

### Cross-sectional study

The main objectives for our cross-sectional study are as follows:

- To calculate the prevalence of reviews that reported that they contacted studies to obtain additional information
- To calculate the prevalence of reviews that reported the number of all contacted studies
- To calculate the prevalence of reviews that reported that all studies with at least one “Unclear” (as a result of missing or insufficient information) risk of bias score were contacted
- To calculate the prevalence of reviews that reported the number of all replying studies
- To calculate the prevalence of replying studies among the contacted studies
- To calculate the prevalence of reviews that reported what information data was (were) obtained from each of the replying studies
- To calculate the prevalence of reviews that reported the consequences of each of the obtained information data that was (were) obtained from each of the replying studies
- To calculate the prevalence of eligible primary studies with at least one domain scored as “Unclear” risk of bias

### Survey

The main objectives for our survey are as follows:

- To calculate the prevalence of reviews that contacted studies to obtain additional information
- To calculate the prevalence of reviews in which all contacted studies had valid contact data
- To quantify the consequences of contacting studies on risk of bias scores, GRADE scores [10], and on the primary and secondary outcomes of the review

### Cross-sectional study versus the survey

The following objective was defined:

- To quantify the prevalence of reviews that reported in both the cross-sectional study and in the survey that studies were contacted to obtain additional information

## Methods

We adopted the Preferred Reporting Items for Systematic review and Meta-analysis Protocols (PRISMA-P) 2015 statement and the Strengthening the Reporting of Observational Studies in Epidemiology (STROBE) statement as the frameworks for reporting this protocol, because these guidelines cover most of our planned methods [14, 35–37]. Our procedures for developing this protocol were conducted by three operators (RMR, LL, and NDG). Differences between this protocol and the final manuscript will be presented in the completed research study together with the rationale for these changes.

**Fig. 1:**
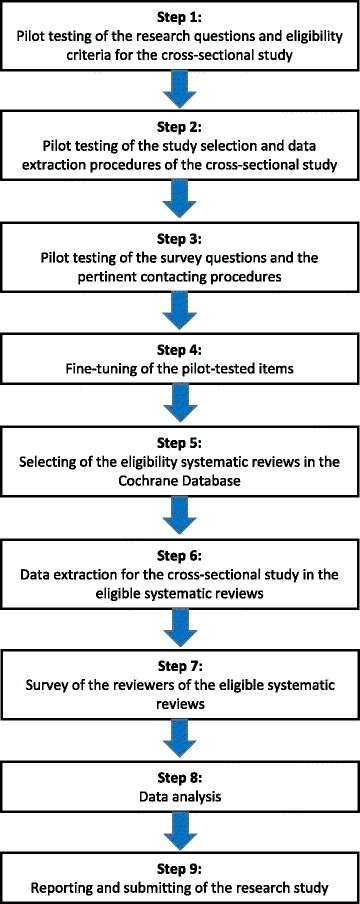
Flow diagram of the research methods

Figure 1 summarizes the flow of the methods in this research study. We first pilot tested the following items on 2 months (July and August 2015) of eligible new Cochrane intervention reviews: (1) our research questions and eligibility criteria for the cross-sectional study, (2) the study selection and data extraction procedures for the cross-sectional study, (3) the survey questions and the pertinent contacting procedures (steps 1–3). These items were subsequently fine-tuned (step 4). Our pilot tests are summarized in Additional file 1. For step 5, we will select eligible systematic reviews in the Cochrane Database. The same selected eligible reviews will be used for both the cross-sectional study and the survey. To avoid the risk of foreknowledge of outcomes, we will complete all data extraction for the cross-sectional study (step 6) prior to conducting the survey (step 7). To avoid bias during data analyses (step 8), we will use Excel files that conceal all reference to the origin of the data, i.e., files without the titles of the reviews and the names and emails of the authors. In the following sections, we will first present our methods for the cross-sectional study and then our methods for the survey.

## Methods for the cross-sectional study

### Eligibility criteria

Type of studies:

- Only Cochrane systematic reviews will be included. These reviews will be selected, because they are considered the reference standard for conducting and reporting such research studies [38].
- We want to include a full year of Cochrane systematic reviews. Only Cochrane reviews published in 2016 are eligible, because this is the most current complete year.
- In 2011, Cochrane working groups started to develop the “Methodological Expectations of Cochrane Intervention Reviews (MECIR)” standards to maintain the high quality of these reviews [9]. The MECIR standards have seen various updates, and until October 2016, they were specifically designed for new Cochrane intervention reviews and were not applicable for “updates” of Cochrane reviews [9, 39-41]. In this study, we will only include new Cochrane intervention reviews published in 2016 and will exclude updates, because this change of the MECIR standard was relatively recent and including updates of reviews published prior to October 2016 could introduce additional heterogeneity and bias.
- Manuscripts labeled in the Cochrane library as “Protocol,” “Withdrawn Protocols,” “Methodology,” “Diagnostic,” “Overview,” “Prognosis,” “Qualitative,” “New search,” “Update,” and “Withdrawn” will be excluded. Earlier published reviews that were later split into two or more new reviews will also be excluded.
- Reviews that included one or more primary studies will be eligible. Empty reviews [42], i.e., reviews that did not identify eligible studies for the research question will be excluded, because our study will focus on contacting authors of eligible studies and not on contacting authors of potentially eligible studies (see interventions).
- Eligibility criteria will not be applied for specific research designs, participants, interventions, comparators, outcomes, endpoints, or settings in the primary studies of the eligible systematic reviews.
- Since we will also quantify “Unclear” risk of bias scores in the eligible primary studies, we will exclude reviews that do not present the pertinent risk of bias tables with these scores.

Interventions:

- Eligible interventions refer to contacting by reviewers of the authors of eligible primary studies included in the review to obtain additional research data on these studies (e.g., information on unreported or missing data, individual patient data, clarification of research methods, and bias issues).
- Non-eligible interventions are (1) contacting by reviewers of authors of primary research studies to verify their eligibility for the review, (2) contacting by reviewers of researchers or research sponsors to get information on additional unknown completed or ongoing studies, and (3) contacting by reviewers of research sponsors to obtain additional research data of the eligible primary studies. We will exclude these interventions, because it will make our cross-sectional study too broad and too difficult to handle
- We expect that most of the contacting procedures are done through emails, but any method for contacting authors such as sending letters, telephoning, faxing, or visiting investigators of primary research studies will also be eligible.

Outcomes:

- Our statistics to address our primary objectives are based on data on contacting and replying of original investigators of eligible studies. Confusion on calculating these statistics could arise when more than one author was contacted or more than once. For the interventions in this study, we will therefore use the terms “contacting studies” and “replying studies”. We will consider each “contacted study” or each “replying study” as a single entity, even when several of its authors were contacted or replied once or several times.

### Information sources and search strategy

The Cochrane Database of Systematic Reviews (CDSR) will be hand-searched for eligible systematic reviews published in this database for the year 2016 [43].

### Study records

#### Data management

To apply our study selection procedures consistently and to reduce inter-examiner disagreements, we consulted the guidelines of the Cochrane Handbook for Systematic Reviews of Interventions and the PRISMA-P 2015 statement [8, 35, 36].

#### Selection of studies

Selection of studies is based on the following:

- Prior to the formal study selection, we conducted pilot tests to fine-tune these procedures and to calibrate reviewers (Fig. 1)(Additional file 1).
- Two reviewers (RMR and LL) will conduct the formal searches for eligible reviews published in 2016 in The Cochrane Database of Systematic Reviews (CDSR) [43]. We will search under the heading “Reviews” and will only consider intervention reviews.
- We will also check the “History” section and the section “References to other published versions of this review” of each potentially eligible intervention reviews to exclude potentially “mislabeled” reviews.
- Disagreements on eligibility of studies between authors will be resolved through discussions. A methodologist (NDG) will be consulted to resolve persisting disagreements.
- All study selection steps will be presented in a PRISMA flow diagram [44]. References of all included studies will be listed in a table. References to excluded studies will be listed separately in a table with the rationale for their exclusion.

#### Data extraction and management

We will download all eligible reviews as PDFs and will divide them according to the month of publication. Our data extraction and management procedures consist of various steps which are explained under here.

Defining “contacting studies”:

- “Contacting studies” refers to studies that are contacted to obtain additional information on an eligible article (e.g., unreported or missing data, individual patient data, clarification of research methods, and risk of bias issues).
- The authors that are contacted in the eligible studies of the reviews can be any author of these studies.

Defining search terms:

- To identify pertinent subject headings and keywords, we applied the following protocol: (1) All new intervention reviews published in the July 2015 Cochrane Library that explicitly described the methods and outcomes of contacting authors were selected by two review authors (RMR and LL). These reviews were then hand-searched to detect and fine-tune pertinent subject headings associated with procedures for contacting studies; (2) synonyms, acronyms, related terms, abbreviations, and variant spellings of these search terms were also identified. Thesauri of databases were consulted for this purpose.
- These searches identified a variety of search terms and their respective grammatical derivatives which are presented in Additional file 2.

Developing data extraction strategies:

- We adopted the Adobe Acrobat protocol for searching and marking multiple words in a PDF [45].
- We used this protocol to assist with our hand searching of the pertinent data items in the eligible systematic reviews. We conducted three different tests to validate these data extraction strategies (Additional file 1).
- We will merge all PDFs of the eligible reviews into 12 single binder PDFs. Each binder PDF will contain all eligible reviews for a specific month of the year 2016 [46]. This method was chosen, because it will reduce the risk of introducing error, because multiple search terms do not have to be re-typed in the Adobe Acrobat search box for each eligible study. Merging the PDFs of the entire year of 2016 into one single PDF was avoided, because such a document will become difficult to handle.

**Fig. 2:**
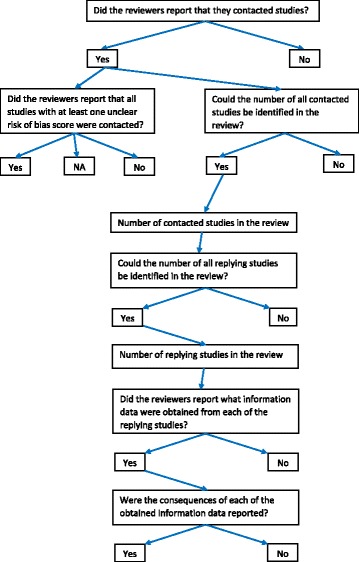
Flow diagram of the research questions on contacting of authors in the cross-sectional study. NA not applicable

Developing data collection forms and defining data items:

- The new Cochrane intervention reviews published in July and August 2015 were used to (1) define a broad-spectrum of data items, (2) develop and fine-tune data collection forms, and (3) pilot-test inter-operator differences in the data extraction procedures (Additional file 1).
- The pilot tested data collection forms are presented in Additional file 3 and will be transferred to an Excel form for the actual data extraction.
- The criteria for completing these forms were defined in detail in order to reduce the need of revisiting selected papers and to improve reproducibility and inter-operator accuracy [36, 47].
- Our questions on contacting of authors for the cross-sectional study are presented in Table 1, and Fig. 2 presents the flow diagram of these questions.

**Table 1:** Questions on contacting of authors for the cross-sectional study

Section	Questions and answers and criteria for addressing questions
Reporting on contacting of studies	Question 1. Did the reviewers report that they contacted studies?
	Answer 1. Yes/no
	Criteria for addressing question 1:
	Yes: When the reviewers report that they contacted one or more studies to obtain additional information. “Yes” is also scored when reviewers reported that they wanted to contact one or more of these studies, but contact information could not be obtained.
	No: When the reviewers did not report whether studies were contacted to obtain additional information.
	No: When reviewers report that they did not contact studies to obtain additional information.
	“No” is also scored when reviewers describe that they planned to contact studies in the methods section, but do not further report on contacting studies.
	“No” is also scored when in the section “Contribution of Authors” specific reviewers are linked to contacting authors without further specification, but in the review itself there is no reported proof that studies were actually contacted. The rationale for this “No” score is that such linking could indeed refer to contacting of studies to obtain additional information (e.g., missing data and risk of bias), but could also refer to contacting of authors to assess the eligibility of studies or contacting of authors to identify additional or ongoing studies.
Reporting on contacting of studies with “Unclear” risk of bias domains^a^	Question 2. Did the reviewers report that all studies with at least one “Unclear” (as a result of missing or insufficient information) risk of bias score were contacted?
	Answer 2. Yes/no/not applicable (NA)
	Criteria for addressing question 2:
	Yes: Studies were contacted and all studies with at least one “Unclear” (as a result of missing or insufficient information) risk of bias score were contacted. We will still score “Yes” when reviewers reported that they wanted to contact all studies with “Unclear” (as a result of missing or insufficient information) risk of bias scores, but contact information could not be obtained for one or more of these studies.
	No: Studies were contacted, but not all studies with at least one “Unclear” (as a result of missing or insufficient information) risk of bias score were contacted.
	NA: Studies were contacted, but “Unclear” (as a result of missing or insufficient information) risk of bias scores were not assigned to any of the domains of the included studies.
Reporting all contacted studies	Question 3. Could the number of all contacted studies be identified in the review?
	Answer 3. Yes/no
	Criteria for addressing question 3:
	Yes: When the number of all contacted studies could be identified in the review. We still scored “Yes” when reviewers reported that they wanted to contact one or more of these studies, but contact information could not be obtained.
	No: When the number of all contacted studies could not be identified in the review.
	No: When one or more studies have been contacted, but it was impossible to identify the exact total number of studies that were contacted.
Number of contacted studies	Question 4. What is the number of contacted studies in the review?
	Answer 4. Present the number of contacted studies in the review.
	Criteria for addressing question 4:
	Only the actual number of studies that was contacted will be scored. Studies that were not contacted because contact information was not available will not be included in this number.
Reporting all replying studies	Question 5. Could the number of all replying studies be identified in the review?
	Answer 5. Yes/no
	Criteria for addressing question 5:
	Yes: When the number of all replying studies could be identified in the review.
	No: When the number of all replying studies could not be identified (e.g., as a result of unclear reporting) in the review.
	No: When one or more contacted studies replied, but it was impossible to identify the exact total number of studies that replied.
Number of replying studies	Question 6. What is the number of contacted studies in the review that replied?
	Answer 6. Present the number of studies that replied.
	Criteria for addressing question 6:
	The actual number of studies that replied will be scored.
Reporting on obtained additional information data	Question 7. Did the reviewers report what information data was(were) obtained from each of the replying studies?
	Answer 7. Yes/no
	Criteria for addressing question 7:
	Yes: The reviewers reported what information data was(were) obtained from each of the replying studies.
	Yes: The reviewers reported that the replying studies explained that they could not provide the requested data.
	No: The reviewers did not report what information data was(were) obtained from each of the replying studies.
	No: The reviewers reported that information data was(were) obtained from the replying studies, but this was partially reported or it was unclear what these data were.
Reporting on the consequences of obtained additional information data	Question 8. Were the consequences (e.g., modified statistics and risk of bias or GRADE scores) of each of the obtained information data reported?
	Answer 8. Yes/no
	Criteria for addressing question 8:
	Yes: The review reported the consequences of each of the obtained information data.
	No: The review did not report the consequences of any of the obtained information data.
	No: The review reported the consequences of some obtained information data, but not for each of the obtained information data.

^a^Higgins et al. [47] divide the definition of “Unclear” risk of bias in three subgroups: Studies are assessed as at unclear risk of bias (1) when too few details are available to make a judgement of “high” or “low” risk; (2) when the risk of bias is genuinely unknown despite sufficient information about the conduct; or (3) when an entry is not relevant to a study (for example because the study did not address any of the outcomes in the group of outcomes to which the entry applies). In this cross-sectional study, we only refer to contacting of authors for the first subgroup of the definition of “Unclear” risk of bias by Higgins et al. [47].

Extracting data from reports:

- Because our pilot testing showed the reliability of the Adobe Acrobat protocol for the identification of the pertinent multiple search terms, we will implement this search tool for all our data extraction procedures during our manual searches [45].
- This protocol will not be applied to just the text of the eligible systematic reviews, but will cover the entire Cochrane review including all figures, tables, and references.
- All research data will be extracted independently by two reviewers (RMR and LL). Disagreements on extracted data between these operators will be resolved through discussion. A third operator (NDG) will be consulted in the case of persisting disagreements.
- To facilitate these discussions, both reviewers will highlight all pertinent items on contacting studies in “yellow” in the binder PDFs.
- When during the data extraction procedures additional relevant data items are identified, we will include these items in the data collection forms and explain the rationale for collecting them and will report this information in the section “Differences between protocol and review”.

## Methods for the survey

Our cross-sectional pilot study of 2 months of new Cochrane intervention reviews (July and August 2015) showed that various items could not be extracted from these reviews, because they were underreported. We therefore decided to include a survey to extract these underreported items. We will use the same reviews that are eligible for the cross-sectional study, i.e., new Cochrane intervention reviews published in 2016 also for our survey. For developing our survey questions, we consulted the guidelines outlined by Fowler [48] and the Checklist for Reporting Results of Internet E Surveys (CHERRIES) [49, 50]. The survey was pilot-tested on the same 2 months of new Cochrane intervention reviews as those used for the cross-sectional study.

Our survey questions on contacting authors by systematic reviewers are presented in Table 2. We will use Google Forms as the platform for our survey [51]. We created an introductory email for the survey and a landing page with an abridged protocol to provide the surveyees with additional information on our survey (Additional file 4). We consulted our published protocols for contacting authors of primary research studies to fine-tune our contacting procedures [52–55].

**Table 2:** Questions on contacting of authors for the survey

Section	Questions and answers and criteria for addressing questions
Title of the systematic review	Question 1. Could you please insert the title of your review?
	(Please copy and paste your review title from our email)
	Answer 1. Insert title: ……………………………………………………………………………………..
Contacting of the eligible included studies for additional information	Question 2. Did you contact any of the study authors of the eligible studies in your review to obtain additional information (e.g., issues on risk of bias and unclear or missing data) on these included studies?
	Please assess carefully the possible answers to this question:
	Yes: If you contacted (through mail, telephone etc.) one or more authors of the eligible studies in your review to obtain additional information on these included studies (e.g., issues on risk of bias, unclear, or missing data).
	No: When authors were not contacted or when authors were contacted for other reasons e.g., to verify potential eligibility of studies or to obtain information on unknown completed or ongoing studies.
	Answer 2. Yes or no
Valid contact data	Question 3. Had all contacted studies valid contact data?
	Please assess carefully the possible answers to this question:
	Valid contact data refers to having one or more of the following functioning contact data, i.e., email addresses, telephone numbers, fax numbers, and postal addresses.
	Answer 3. Yes or no
Effect on risk of bias scores	Question 4. Were one or more risk of bias scores in your review modified as a result of the information obtained from contacted studies?
	Please assess carefully the possible answers to this question:
	Yes: One or more risk of bias scores in your review were modified as a result of the information obtained from the contacted studies.
	No: None of the risk of bias scores in your review were modified as a result of the information obtained from the contacted studies.
	Not applicable: Risk of bias was not assessed in your review or this question could not be addressed for other reasons.
	Answer 4. Yes, no, not applicable
Effect on the GRADE score	Question 5. Were GRADE scores for one or more outcomes modified as a result of the information obtained from the contacted studies?
	Please assess carefully the possible answers to this question:
	Yes: GRADE scores for one or more outcomes were modified as a result of the information obtained from the contacted studies.
	No: GRADE scores for one or more outcomes were not modified as a result of the information obtained from the contacted studies.
	Not applicable: GRADE scores were not assessed in your review or this question could not be addressed for other reasons.
	Answer 5. Yes, no, not applicable
Effect on the summary primary or secondary outcomes	Question 6. Were any of the summary primary or secondary outcomes of the review modified as a result of the information obtained from the contacted studies?
	Please assess carefully the possible answers to this question:
	Yes: One or more summary primary or secondary outcomes of the review were modified as a result of the information obtained from the contacted studies.
	No: None of the summary primary or secondary outcomes of the review were modified as a result of the information obtained from the contacted studies.
	Answer 6. Yes or no
Effect on the summary effect size of the primary outcome	Question 7. How was the summary effect size of the primary outcome modified as a result of the information obtained from the contacted studies?
	Please assess carefully the possible answers to this question:
	Increased: The summary effect size of the primary outcome increased as a result of the information obtained from the contacted studies.
	Decreased: The summary effect size of the primary outcome decreased as a result of the information obtained from the contacted studies.
	Unchanged: The summary effect size of the primary outcome did not change as a result of the information obtained from the contacted studies.
	Not applicable: The summary effect size of the primary outcome was not measured in your review or this question could not be addressed for other reasons.
	Answer 7. Increased, decreased, unchanged, not applicable
Optional question	Question 8. Could you please describe some issues that you want to share with us regarding contacting of study authors of the eligible studies included in your review?
	Answer 8. I have the following issues to share:
	I have no further issues to share.

Our procedures for conducting our survey are presented as a step-by-step process:

- Step 1. The titles, names of the authors, and the contact addresses of the eligible reviews will be extracted and copied and pasted into an Excel data sheet.
- Step 2. Our survey will be sent with an introductory email to the contact address of each eligible review. We will only send the survey to the contact address of the eligible reviews and not to any of the co-authors. When more than one email address was provided for the same contact author, we will send the survey to both addresses. In the initial email, authors will answer our survey through a link presented in this email. In all reminder emails, we give authors the option to either answer the survey through a link or simply by completing the survey presented at the end of the introductory email.
- Step 3. In the first week of our survey, we will send our initial email and our first reminder email (in the case that no response will be received to our initial email). We will send this first reminder email after a minimum of three business days [30].
- Step 4. When still no response will be received, we will continue to send reminder emails during the following 2 weeks (two reminders per week). In each reminder email, we will indicate the current response rate to our survey. After a maximum of five reminder emails, we will stop the survey. In our fifth reminder email, we will indicate that it is the last email for our survey.
- When automated responses like “out of office,” “vacation leave,” maternity leave, and study leave will be received, we will apply our protocol at the author’s return.

### Primary outcome and power calculation

Our primary outcome for this research study will be the prevalence of reviews that reported that eligible primary studies were contacted to obtain additional information. For this statistic, the following calculation is made: the number of reviews that reported that they contacted studies to obtain additional information/the total number of eligible reviews. To calculate the required sample size, we conducted a power calculation using software developed by EpiTools epidemiological calculators [56]. We first conducted a pilot test on all eligible Cochrane systematic reviews published in July and August 2015 to identify the estimated prevalence for the primary outcome. This pilot study showed that 40 of 52 (76.92%) eligible reviews were contacted to obtain additional information. EpiTools calculated a sample size of 273 for the estimated prevalence of 0.7692 with a 0.95 confidence level (desired precision of estimate 0.05) [56].

Since 2 months (July and August 2015) of new Cochrane intervention reviews provided 52 eligible reviews, this would account for 26 reviews per months. We decided to include a complete year (2016) of new Cochrane intervention reviews, i.e., 312 reviews (12 × 26). When 12 months of new Cochrane intervention reviews will not provide the required sample size of 273 reviews, we will include subsequent months of eligible reviews, i.e., from January 2017 onwards until the desired sample size will be reached.

### Outcome measures and statistical analysis

The planned outcomes for the cross-sectional study and the survey will be presented in summary of findings tables (Additional file 5). We will also compare outcomes measured in the cross-sectional study with those in the survey (Additional file 5). Outcomes that will be introduced or eliminated post hoc will be reported in the completed study together with the rationale for including or excluding them. Stata software (Stata Corporation, College Station, Texas, USA) version 15 will be used for the statistical analysis [57]. Prevalence data will be reported with their 0.95 confidence levels.

### Differences between the protocol and the completed study

Differences between the protocol and the completed study will be presented, and the rationale for these modifications will also be reported. The consequences of these modifications on the magnitude, direction, and the validity of outcomes will also be given [58].

## Discussion

Systematic reviewers address focused questions by appraising and synthesizing all relevant research evidence. The quality of these reviews strongly depends on the quality of the reporting of the eligible primary studies. Contacting authors of these studies to obtain additional information could be an important research procedure for dealing with suboptimal reporting. This study will explore issues such as (1) how reviewers report on contacting of authors of eligible studies in the review and the outcomes of these procedures, (2) the validity of contact data in contacted studies, (3) how contacted investigators reply, (4) how reviewers report on the obtained additional data, (5) what the consequences of these contacting procedures are for outcomes and quality scores in the review, (6) the differences between outcomes reported in the review with those recorded in the survey, and (7) the prevalence of primary studies with at least one domain scored as “Unclear” risk of bias.

Positive findings are important, because little has been published on these research questions and such outcomes could address some knowledge gaps and could quantify the importance of contacting of authors. However, negative outcomes are expected, because our pilots test showed (1) that procedures of contacting authors and the consequences are underreported in systematic reviews of interventions and (2) that contacted authors of primary studies reply poorly to reviewers. These negative findings could show the need for developing guidelines for reporting and conducting of contacting procedures of primary research studies and their outcomes in systematic reviews. These findings could direct research on this topic and could also show the need to keep contact data of investigators up to date in a central register. Better methods of conducting and reporting of communication between systematic reviewers and authors of primary research studies could lead to a better understanding of what was done in the eligible studies and could ultimate improve the quality and the trustworthiness of the review and its reproducibility. Patients, clinicians, researchers, guideline developers, research sponsors, and the general public will all be beneficiaries.

## Additional files

Additional file 1: Pilot tests. (DOCX 42 kb)

Additional file 2: Search terms and their derivatives. (DOCX 37 kb)

Additional file 3: Data collection forms for the cross-sectional study. (DOCX 43 kb)

Additional file 4: Introductory initial email and abridged protocol for the survey. (DOCX 46 kb)

Additional file 5: Summary of findings tables. (DOCX 42 kb)

## Abbreviations

EQUATOR Network: Enhancing the Quality and Transparency Of health Research Network
MECIR: Methodological Expectations of Cochrane Intervention Reviews
