# Supplementary material for: Contacting of authors by systematic reviewers: protocol for a cross-sectional study and a survey
Source: Syst Rev. 2017 Dec 8;6:249. doi: 10.1186/s13643-017-0643-z (PMC5721423; doi:10.1186/s13643-017-0643-z)
Supplement: Supplementary file 1 — Pilot tests. (DOCX 42 kb) [file 13643_2017_643_MOESM1_ESM.docx]

**Additional file 1. Pilot tests for the cross-sectional study and survey**

- All new Cochrane intervention reviews published in the months July and August 2015 were used for the pilot tests of the cross-sectional study.
- Two operators (RMR and LL) conducted these tests for the cross-sectional study.
- We pilot tested the following items: (1) A wide variety of research questions (2) criteria for addressing these questions (3) the eligibility criteria for the review (4) procedures for selecting studies and (5) data extraction strategies.
- The findings of these pilot tests were subsequently used to fine-tune these items. These tests were also conducted to calibrate the 2 operators for the various research procedures.
- To assess the validity of our data Adobe protocol for searching and marking multiple words in PDFs [45] we conducted three different pilot tests (1) We created a PDF that included sentences with all our search terms, including their derivatives and respective hyphenations. We then assessed whether our Adobe Acrobat protocol could identify all of these items (2) We also applied this protocol to the new Cochrane intervention reviews published in July 2015. We assessed whether the Adobe control F function would identify the same search terms in the PDFs as those highlighted by our Adobe Acrobat protocol. (3) We also assessed whether our Adobe Acrobat protocol could identify the pertinent search terms of interest as precise or better than our hand-searching procedures. Again, we pilot tested this hypothesis on the new Cochrane intervention reviews published in July 2015. The accuracy of our Adobe Acrobat protocol was confirmed by these 3 test.
- We also used all new Cochrane intervention reviews published in the months July and August 2015 to pilot test our survey questions and procedures for conducting the survey.
