# Supplementary material for: Contacting of authors by systematic reviewers: protocol for a cross-sectional study and a survey
Source: Syst Rev. 2017 Dec 8;6:249. doi: 10.1186/s13643-017-0643-z (PMC5721423; doi:10.1186/s13643-017-0643-z)
Supplement: Supplementary file 2 — Search terms and their derivatives. (DOCX 37 kb) [file 13643_2017_643_MOESM2_ESM.docx]

**Additional file 2. Search terms and their derivatives**

**Search terms and their derivatives**

| **Search terms and their derivatives** | **Search terms for searching multiple words in a PDF** |
| --- | --- |
| “additional”, “addition” | ADD, Add, add |
| “add”, “adds”, “adding”, “added” | ADD, Add, add |
| “answer”, “answers”, “answering”, “answered” | ANSWER, Answer, answer |
| “ask”, “asks”, “asking”, “asked” | ASK, Ask, ask |
| “attempt”, “attempts”, “attempting”, “attempted” | ATTEMP, Attemp, attemp |
| “author”, “authors” | AUTHOR, Author, author |
| “communicate”, “communicates”, “communicating”, “communicated”, communicational” | COMMUNICAT, Communicat, communicat |
| “consult”, “consults”, “consulting”, “consulted” | CONSULT, Consult, consult |
| “contact”, “contacts”, “contacting”, “contacted” | CONTACT, Contact, contact |
| “correspondence”, “correspond”, “corresponds”, “corresponding”, “corresponded”, “corresponder”, corresponders” | CORRESPOND, Correspond, correspond |
| Data | DATA, Data, data |
| “eligibility”, “eligibilities”, “eligible” | ELIGIB, Eligib, eligib |
| “email”, “emails”, “emailing”, “emailed” | MAIL, Mail, mail |
| “information” | INFO, Info, info |
| “inform”, “informs”, “informing”, “informed” | INFO, Info, info |
| “investigate”, “investigates”, “investigating”, “investigated”, “investigator”, “investigators”, “investigation”, “investigations” | INVESTIGAT, Investigat, investigat |
| “letter”, “letters” | LETTER, Letter, letter |
| “mail”, “mails”, “mailing”, “mailed” | MAIL, Mail, mail |
| “obtain”, “obtains”, “obtaining”, “obtained” | OBTAIN, Obtain, obtain |
| “question”, “questions”, “questioning”, “questioned” | QUEST, Quest, quest |
| “questionnaire”, “questionnaires” | QUEST, Quest, quest |
| “quest”, “quests”, “questing”, “quested” | QUEST, Quest, quest |
| “reply”, “replies”, “replying”, “replied”, “replier”, “repliers” | REPL, Repl, repl |
| “research”, “researches”, “researching”, “researched”, “researcher”, “researchers” | RESEARCH, Research, research |
| “response”, “responses”, “respond”, “responds”, “responding”, “responded”, “responder”, “responders” | RESPON, Respon, respon |
| “request”, “requests”, “requesting”, “requested” | QUEST, Quest, quest |
| “retrieve”, “retrieves”, “retrieving”, “retrieved” | RETRIEV, Retriev, retriev |
| “telephone”, “telephones”, “telephoning”, “telephoned”, “phone”, “phones”, “phoning”, “phoned” | PHON, Phon, phon |
| “trial”, “trials”, “trialist”, “trialists” | TRIAL, Trial, trial |
| “write”, “writes”, “writing”, “wrote”, “written” | WRIT, Writ, writ  WROT, Wrot, wrot |
