# Supplementary material for: Contacting of authors by systematic reviewers: protocol for a cross-sectional study and a survey
Source: Syst Rev. 2017 Dec 8;6:249. doi: 10.1186/s13643-017-0643-z (PMC5721423; doi:10.1186/s13643-017-0643-z)
Supplement: Supplementary file 3 — Data collection forms for the cross-sectional study. (DOCX 43 kb) [file 13643_2017_643_MOESM3_ESM.docx]

**Additional file 3. Data collection form for the cross-sectional study***

**Data collection forms for the cross-sectional study**

| **Item Letter** | **Item** | **Description of each item and criteria for scoring each item in the data collection forms** |
| --- | --- | --- |
| A | Month | January through December |
| B | Page number in the binder document | Present the initial page number of each individual systematic review in the binder document. |
| C | Reference | List title |
| D | Review group | Present the pertinent review group. |
| E | Number of eligible studies in the risk of bias table | Present the number of eligible studies in the risk of bias table. |
| F | Did all eligible studies in the review have at least one domain scored as ‘Unclear’ risk of bias in the risk of bias table? | Answer: Yes/No |
| G | # of eligible studies with at least one domain scored as ‘Unclear’ risk of bias in the risk of bias table | Present the number of eligible studies with at least one domain scored as ‘Unclear’ risk of bias. |
| H | Page and potential comment(s) | Present the page(s) that addressed this item.  This information is found in the Risk of bias table.  Present a potential comment on this item. |
| I | Page and potential comment(s) | Present the page(s) that addressed this item.  Present a potential comment on this item. |
| J | Did the reviewers report that they contacted studies? | Answer: Yes/No  Criteria for addressing this question:  Yes: When the reviewers report that they contacted one or more studies to obtain additional information. ‘Yes’ is also scored when reviewers reported that they wanted to contact one or more of these studies, but contact information could not be obtained, e.g., older studies etc.  No: When the reviewers did not report whether studies were contacted to obtain additional information.  No: When reviewers report that they did not contact studies to obtain additional information.  ‘No’ is also scored when reviewers describe that they planned to contact studies in the methods section, but do not further report on contacting studies.  ‘No’ is also scored when in the section ‘Contribution of Authors’ specific reviewers are linked to contacting authors without further specification, but in the review itself there is no reported proof that studies were actually contacted. The rationale for this ‘No’ score is that such linking could indeed refer to contacting of studies to obtain additional information (e.g., missing data, risk of bias etc.), but could also refer to contacting of authors to assess the eligibility of studies or contacting of authors to identify additional or ongoing studies. |
| K | Page and potential comment(s) | Present the page(s) that addressed this item.  Present a potential comment on this item. |
| L | Did the reviewers report that all studies with at least one ‘Unclear’ (as a result of missing or insufficient information) risk of bias score were contacted?** | Answer: Yes/No/Not applicable (NA)  Criteria for addressing this question:  Yes: Studies were contacted and all studies with at least one ‘Unclear’ (as a result of missing or insufficient information) risk of bias score were contacted. We will still score ‘Yes’ when reviewers reported that they wanted to contact all studies with ‘Unclear’ (as a result of missing or insufficient information) risk of bias scores, but contact information could not be obtained for one or more of these studies.  No: Studies were contacted, but not all studies with at least one ‘Unclear’ (as a result of missing or insufficient information) risk of bias score were contacted.  NA: Studies were contacted, but ‘Unclear’ (as a result of missing or insufficient information) risk of bias scores were not assigned to any of the domains of the included studies. |
| M | Could the number of all contacted studies be identified in the review? | Answer: Yes/No  Criteria for addressing this question:  Yes: When the number of all contacted studies could be identified in the review. We still scored ‘Yes’ when reviewers reported that they wanted to contact one or more of these studies, but contact information could not be obtained.  No: When the number of all contacted studies could not be identified in the review.  No: When one or more studies have been contacted, but it was impossible to identify the exact total number of studies that were contacted. |
| N | What is the number of contacted studies in the review | Present the number of studies that was contacted.  Criteria for addressing this question:  Only the actual number of studies that was contacted will be scored. Studies that were not contacted because contact information was not available will not be included in this number.  NA: When the question (M) ‘Could the number of all contacted studies be identified in the review’? was answered with a ‘No’. |
| O | Page and potential comment(s) | Present the page(s) that addressed this item  Present a potential comment on this item. |
| P | Could the number of all replying studies be identified in the review? | Answer: Yes/No/Not Applicable (NA)  Criteria for addressing this question:  Yes: When the number of all replying studies could be identified in the review.  No: When the number of all replying studies could not be identified (e.g., as a result of unclear reporting) in the review.  No: When one or more contacted studies replied, but it was impossible to identify the exact total number of studies that replied.  NA: When the question (M) ‘Could the number of all contacted studies be identified in the review’? was answered with a ‘No’. |
| Q | What is the number of contacted studies in the review that replied? | The number of studies that replied.  Criteria for addressing this question:  The actual number of studies that replied will be scored.  NA: When the question (M) ‘Could the number of all contacted studies be identified in the review’? was answered with a ‘No’.  NA: When the question (P) ‘Could the number of all replying studies be identified in the review’ was answered with a ‘No’. |
| R | Page and potential comment(s) | Present the page(s) that addressed this item.  Present a potential comment on this item. |
| S | Did the reviewers report what information data was (were) obtained from each of the replying studies? | Answer: Yes/No/Not Applicable (NA)  Criteria for addressing this question:  Yes: The reviewers reported what information data was (were) obtained from each of the replying studies.  Yes: The reviewers reported that the replying studies explained that they could not provide the requested data.  No: The reviewers did not report what information data was (were) obtained from each of the replying studies.  No: The reviewers reported that information data was (were) obtained from the replying studies, but this was partially reported or it was unclear what these data were.  NA: When the question (M) ‘Could the number of all contacted studies be identified in the review’? was answered with a ‘No’.  NA: When the question (P) ‘Could the number of all replying studies be identified in the review’ was answered with a ‘No’. |
| T | Page and potential comment(s) | Present the page(s) that addressed this item  Present a potential comment on this item. |
| U | Were the consequences (e.g., modified statistics, risk of bias or GRADE scores etc.) of each of the obtained information data reported? | Answer: Yes/No/Not Applicable (NA)  Criteria for addressing this question:  Yes: The review reported the consequences of each of the obtained information data.  No: The review did not report the consequences of any of the obtained information data.  No: The review reported the consequences of some obtained information data, but not for each of the obtained information data.  NA: When the question (M) ‘Could the number of all contacted studies be identified in the review’? was answered with a ‘No’.  NA: When the question (P) ‘Could the number of all replying studies be identified in the review’ was answered with a ‘No’.  NA: When the question (S) ‘Did the reviewers report what information data was (were) obtained from each of the replying studies’ was answered with a ‘No’. |
| V | Page and potential comment(s) | Present the page(s) that addressed this item  Present a potential comment on this item. |
| W | General Comments | Write a comment about the study that summarizes key issues that should be helpful when discussing the study with the other operator(s). Make reference to the specific pages. |

*Page numbers of extracted items are listed according to their page number in the review and not according to the number of the page in the binder document.

**Higgins et al. [47] divide the definition of ‘Unclear’ risk of bias in 3 subgroups: (1) ‘Studies are assessed as at unclear risk of bias when too few details are available to make a judgement of ‘high’ or ‘low’ risk; (2) when the risk of bias is genuinely unknown despite sufficient information about the conduct; or (3) when an entry is not relevant to a study (for example because the study did not address any of the outcomes in the group of outcomes to which the entry applies). In this cross-sectional study we only refer to contacting of authors for the first subgroup of the definition of ‘Unclear’ risk of bias by Higgins et al. [47]
