# Supplementary material for: Contacting of authors by systematic reviewers: protocol for a cross-sectional study and a survey
Source: Syst Rev. 2017 Dec 8;6:249. doi: 10.1186/s13643-017-0643-z (PMC5721423; doi:10.1186/s13643-017-0643-z)
Supplement: Supplementary file 4 — Introductory initial email and abridged protocol for the survey. (DOCX 46 kb) [file 13643_2017_643_MOESM4_ESM.docx]

**Additional file 4. Introductory initial email and abridged protocol for the survey**

**Introductory initial email for the survey**

| \| **Review Methods Research** \| \| --- \|   **Contacting of authors by systematic reviewers**  Dear Professor …………,  We would like to invite you to take part in a survey to find out how contacted authors of eligible studies in systematic reviews respond to reviewers.  We thank you a priori for taking the time for completing this survey. Patients, clinicians, and researchers will all benefit enormously from your input!  Your review published in ……. 2016 in the Cochrane Database with the title ‘…………………’ has been selected for this survey.  The survey consists of **8 simple questions**, which have been previously tested and will take roughly **2 minutes** to complete.  Cochrane Intervention Reviews have been selected to be included in a large scale secondary analysis of the literature. Our scope is not to draw any conclusions about your results or to scrutinize your work in any way.  **For taking part in this survey, click on the following link:…………..**  **For additional information on the survey protocol, click on the following link: ……**    We thank you again for your important contribution to this research project.  Yours sincerely,  Reint Meursinge Reynders^1^ DDS, MSc (Evidence-based health care), MS (Oral biology), PhD  Nicola Di Girolamo^2^ DMV, MSc (Evidence-based health care), PhD, DECZM(HERP), EBMVet  ^1^ Department of oral and maxillofacial surgery, Academic Medical Center, University of Amsterdam, Meibergdreef 9, 1105 AZ Amsterdam, The Netherlands.  ^2^ EBMVet, Via Sigismondo Trecchi 20, Cremona CR 26100, Italy.  **Please contact** [reviewmethods@amc.uva.nl](mailto:reviewmethods@amc.uva.nl) or [r.a.meursingereynders@amc.uva.nl](mailto:r.a.meursingereynders@amc.uva.nl) if you encounter any problems with this survey. |
| --- | --- |

**Abridged protocol for the survey**

| **Background and objectives**  Synthesizing the outcomes of poorly reported studies can pose a serious threat to the validity of systematic reviews and healthcare guidelines (Bleakley 2008, Gordon 2011, Kirkham 2010, Meuffels 2011, Nolte 2004). The Cochrane Collaboration therefore recommends reviewers to contact the original investigators of eligible studies and request additional information on poorly reported items (Higgins 2011, Higgins 2016). This survey assesses the following key issues: (1) whether reviewers contacted authors of eligible primary studies to obtain additional information on such poorly reported items (2) the consequences of the obtained additional information for risk of bias scores, GRADE scores, primary and secondary outcomes, and the summary effect size.  **Methods**  **Pilot testing**  All our research methods have been pilot-tested a priori on 2 months of New Cochrane intervention reviews.  **Key eligibility criteria:**  ***Studies:*** New Cochrane Intervention Reviews (MECIR) published in 2016 (Higgins 2016).  ***Interventions:*** Eligible interventions refer to contacting by reviewers of the authors of eligible primary studies included in the review to obtain additional research data on these studies (e.g., information on unreported or missing data, individual patient data, clarification of research methods, bias issues etc.). Non-eligible interventions are contacting of studies by reviewers for other reasons e.g., to verify potential eligibility of studies or to obtain information on unknown completed or ongoing studies.  **Searching for studies:** The Cochrane Database of Systematic Reviews (CDSR) will be hand-searched for eligible systematic reviews published in this database for the year 2016 (CDSR 2017). Searching for eligible studies will be conducted by the same 2 operators independently.    **Procedures for the survey**  In this survey we ask reviewers to answer 8 questions. Replying has been tested previously and will take approximately 2 minutes to complete. Our scope is not to draw any conclusions about the results of the systematic review or to scrutinize the review in any way.  **Importance of the survey**  This survey will quantify the importance of contacting authors of eligible primary studies by systematic reviewers. Its findings could be a stimulus for endorsing and improving these procedures. This could ultimately lead to an increase in the trustworthiness of outcomes presented in systematic reviews. Patients, clinicians, researchers, guideline developers, research sponsors, and the general public will all benefit.  **References**  **Bleakley 2008**  [Bleakley CM](http://www.ncbi.nlm.nih.gov/pubmed/?term=Bleakley%20CM%5BAuthor%5D&cauthor=true&cauthor_uid=18298355), [McDonough SM](http://www.ncbi.nlm.nih.gov/pubmed/?term=McDonough%20SM%5BAuthor%5D&cauthor=true&cauthor_uid=18298355), [MacAuley DC](http://www.ncbi.nlm.nih.gov/pubmed/?term=MacAuley%20DC%5BAuthor%5D&cauthor=true&cauthor_uid=18298355). Some conservative strategies are effective when added to  controlled mobilisation with external support after acute ankle sprain: a systematicreview. [Aust J Physiother.](http://www.ncbi.nlm.nih.gov/pubmed/?term=Bleakley+et+al.+Some+conservative+strategies+are+effective+when+added+to+controlled+mobilisation+with+external+support+after+acute+ankle+sprain%3A+a+systematic+review.) 2008;54(1):7-20.  **CDSR 2017**  Cochrane Database of Systematic Reviews (CDSR). [online] Available from: http://www.cochranelibrary.com/cochrane-database-of-systematic-reviews/(accessed July 28^th^ 2017).  **Gordon 2011**  [Gordon M](http://www.ncbi.nlm.nih.gov/pubmed/?term=Gordon%20M%5BAuthor%5D&cauthor=true&cauthor_uid=21933243), [Findley R](http://www.ncbi.nlm.nih.gov/pubmed/?term=Findley%20R%5BAuthor%5D&cauthor=true&cauthor_uid=21933243). Educational interventions to improve handover in health care: a systematic review. [Med Educ.](http://www.ncbi.nlm.nih.gov/pubmed/?term=Gordon+and+Findlay.+Educational+interventions+to+improve+handover+in+health+care%3A+a+systematic+review.) 2011 Nov;45(11):1081-9.  **Higgins 2011**  Higgins JPT, Deeks JJ. Chapter 7: Selecting studies and collecting data. In: Higgins JPT, Green S (editors), Cochrane Handbook for Systematic Reviews of Interventions Version 5.1.0 (updated March 2011). The Cochrane Collaboration, 2011. [online] Available from: [www.cochrane-handbook.org](http://www.cochrane-handbook.org). (accessed July 28th 2017).  **Higgins 2016**  Higgins JPT, Lasserson T, Chandler J, Tovey D, Churchill R. Methodological Expectations of Cochrane Intervention Reviews (MECIR). Standards for the conduct and reporting of new Cochrane Intervention Reviews, reporting of protocols and the planning, conduct and reporting of updates. Cochrane: London, 2016. [online] Available from: <http://methods.cochrane.org/sites/default/files/public/uploads/mecir_printed_booklet_final.pdf> (accessed July 28th 2017).  **Kirkham 2010**  [Kirkham JJ](http://www.ncbi.nlm.nih.gov/pubmed/?term=Kirkham%20JJ%5BAuthor%5D&cauthor=true&cauthor_uid=20156912), [Dwan KM](http://www.ncbi.nlm.nih.gov/pubmed/?term=Dwan%20KM%5BAuthor%5D&cauthor=true&cauthor_uid=20156912), [Altman DG](http://www.ncbi.nlm.nih.gov/pubmed/?term=Altman%20DG%5BAuthor%5D&cauthor=true&cauthor_uid=20156912), [Gamble C](http://www.ncbi.nlm.nih.gov/pubmed/?term=Gamble%20C%5BAuthor%5D&cauthor=true&cauthor_uid=20156912), [Dodd S](http://www.ncbi.nlm.nih.gov/pubmed/?term=Dodd%20S%5BAuthor%5D&cauthor=true&cauthor_uid=20156912), [Smyth R](http://www.ncbi.nlm.nih.gov/pubmed/?term=Smyth%20R%5BAuthor%5D&cauthor=true&cauthor_uid=20156912), [Williamson PR](http://www.ncbi.nlm.nih.gov/pubmed/?term=Williamson%20PR%5BAuthor%5D&cauthor=true&cauthor_uid=20156912). The impact of outcome reporting bias in randomised controlled trials on a cohort of systematic reviews. [BMJ.](http://www.ncbi.nlm.nih.gov/pubmed/20156912) 2010 Feb 15;340:c365. doi: 10.1136/bmj.c365.  **Meuffels 2011**  [Meuffels DE](http://www.ncbi.nlm.nih.gov/pubmed/?term=Meuffels%20DE%5BAuthor%5D&cauthor=true&cauthor_uid=21678367), [Reijman M](http://www.ncbi.nlm.nih.gov/pubmed/?term=Reijman%20M%5BAuthor%5D&cauthor=true&cauthor_uid=21678367), [Scholten RJ](http://www.ncbi.nlm.nih.gov/pubmed/?term=Scholten%20RJ%5BAuthor%5D&cauthor=true&cauthor_uid=21678367), [Verhaar JA](http://www.ncbi.nlm.nih.gov/pubmed/?term=Verhaar%20JA%5BAuthor%5D&cauthor=true&cauthor_uid=21678367). Computer assisted surgery for knee ligament reconstruction. [Cochrane Database Syst Rev.](http://www.ncbi.nlm.nih.gov/pubmed/21678367) 2011 Jun 15;(6):CD007601.  **Nolte 2004**  [Nolte S](http://www.ncbi.nlm.nih.gov/pubmed/?term=Nolte%20S%5BAuthor%5D&cauthor=true&cauthor_uid=15495131), [Wong D](http://www.ncbi.nlm.nih.gov/pubmed/?term=Wong%20D%5BAuthor%5D&cauthor=true&cauthor_uid=15495131), [Lachford G](http://www.ncbi.nlm.nih.gov/pubmed/?term=Lachford%20G%5BAuthor%5D&cauthor=true&cauthor_uid=15495131). Amphetamines for schizophrenia. [Cochrane Database Syst Rev.](http://www.ncbi.nlm.nih.gov/pubmed/?term=Nolte+et+al.+Amphetamines+for+schizophrenia.+CDSR+2004%5D) 2004 Oct 18;(4):CD004964. |
| --- |
