# Supplementary material for: Contacting of authors by systematic reviewers: protocol for a cross-sectional study and a survey
Source: Syst Rev. 2017 Dec 8;6:249. doi: 10.1186/s13643-017-0643-z (PMC5721423; doi:10.1186/s13643-017-0643-z)
Supplement: Supplementary file 5 — Summary of findings tables. (DOCX 42 kb) [file 13643_2017_643_MOESM5_ESM.docx]

**Additional file 5. Summary of findings tables**

**Summary of findings table for the cross-sectional study**

| **Outcome number** | **Description of outcome measures** | **Type of statistic** |
| --- | --- | --- |
| 1 | Total number of reviews, i.e., refer to the eligible reviews in the cross-sectional study* | Number |
| 2 | The prevalence of each pertinent review groups, e.g., ENT Group, Epilepsy Group, Stroke Group etc. | Prevalence |
| 3 | The prevalence of reviews in which all eligible studies had at least one domain scored as ‘Unclear’ risk of bias. | Prevalence |
| 4 | The prevalence of eligible studies with at least one domain scored as ‘Unclear’ risk of bias. | Prevalence |
| 5 | The prevalence of reviews that reported that they contacted studies to obtain additional information. | Prevalence |
| 6 | The prevalence of reviews that reported that all studies with at least one ‘Unclear’ (as a result of missing or insufficient information) risk of bias score were contacted. | Prevalence |
| 7 | The prevalence of reviews that reported the number of all contacted studies. | Prevalence |
| 8 | The prevalence of reviews that reported the number of all replying studies. | Prevalence |
| 9 | The prevalence of replying studies. | Prevalence |
| 10 | The prevalence of reviews that reported what information data was (were) obtained from each of the replying studies. | Prevalence |
| 11 | The prevalence of reviews that reported the consequences of each of the obtained information data that was (were) obtained from each of the replying studies. | Prevalence |

*The eligible reviews are the same for both the cross-sectional study and the survey

**Summary of findings table for the survey**

| **Outcome number** | **Description of outcome measures** | **Type of statistic** |
| --- | --- | --- |
| 1 | Total number of surveyed reviews, i.e., refer to the eligible reviews for the survey* | Number |
| 2 | The response rate of the surveyed reviews. | Prevalence |
| 3 | The prevalence of surveyed reviews that contacted studies to obtain additional information. | Prevalence |
| 4 | The prevalence of reviews in which all contacted studies had valid contact data (valid email addresses, telephone numbers, postal addresses etc.). | Prevalence |
| 5 | The prevalence of surveyed reviews in which one or more of the risk of bias score(s) were modified as a result of the information obtained from the contacted studies. | Prevalence |
| 6 | The prevalence of surveyed reviews in which the GRADE score was modified as a result of the information obtained from the contacted studies. | Prevalence |
| 7 | The prevalence of surveyed reviews with respectively downgraded, upgraded, and unchanged GRADE scores. | Prevalence |
| 8 | The prevalence of surveyed reviews in which one or more summary primary or secondary outcomes of the review were modified as a result of the information obtained from the contacted studies. | Prevalence |
| 9 | The prevalence of surveyed reviews in which the summary effect size of the primary outcome was modified as a result of the information obtained from the contacted studies. | Prevalence |
| 10 | The prevalence of surveyed reviews with respectively an increased, decreased, and unchanged summary effect size of the primary outcome. | Prevalence |

*The eligible reviews are the same for both the cross-sectional study and the survey

**Summary of findings table for the cross-sectional study versus the survey**

| **Outcome number** | **Description of outcome measures** | **Type of statistic** |
| --- | --- | --- |
| 1 | The number of surveyed reviews that completed the survey. | Number |
| 2 | The prevalence of reviews that reported in both the cross-sectional study and in the survey that studies were contacted to obtain additional information. | Prevalence |
